# A low‐cost field ventilator: An urgent global need

**DOI:** 10.1002/hsr2.349

**Published:** 2021-08-05

**Authors:** Nitish Nachiappan, Jung Moses Koo, Nachiappan Chockalingam, Timothy E Scott

**Affiliations:** ^1^ School of Medicine Imperial College London UK; ^2^ Centre for Biomechanics and Rehabilitation Technologies Staffordshire University Stoke on Trent UK; ^3^ Adult Intensive Care Unit University Hospitals of North Midlands NHS Trust Stoke on Trent UK

**Keywords:** developing countries, intensive care units, LMIC, peripartum period, respiration, artificial, ventilators, mechanical

## Abstract

Modern ventilators are increasingly compact and able to deliver a wide range of ventilator modes and sophisticated monitoring capabilities. However, the global availability of ventilators is woefully short of demand. Data on intensive care units (ICUs), a proxy measure for hospital ventilator capacity in low and middle‐income countries (LMIC's), suggest that capacity is extremely limited where it exists at all. In LMIC's, the four most common indications for mechanical ventilation (MV) in ICUs are post‐surgical care, sepsis, trauma, and maternal peripartum or neonatal complications. A significant majority of these cases can be managed with intervention involving a short course of MV. Widespread and timely access to MV can thus effectively be used to help patients in these settings and improve outcomes. This paper implores this need and highlights the requirements for a low‐cost ventilator or a respiratory support device.

## 1 BACKGROUND

Mechanical ventilation (MV) is a mode of invasive organ support used to maintain adequate lung function in patients with, or at risk of, failing cardiorespiratory function.^1^ From Andreas Vesalius' 16th century accounts of ventilation, negative‐pressure reliant iron lungs in the early 20th century, to the highly sophisticated devices available today, there has been much development of MV throughout history. The search for effective MV for paralytic poliomyelitis patients and Acute Respiratory Distress Syndrome (ARDS) spurred the development of MV technology reliant on positive‐pressure and positive end‐expiratory pressure (PEEP) use.^1^, ^2^

Modern ventilators are increasingly compact and able to deliver a wide range of ventilatory modes with sophisticated monitoring capabilities.^2^ MV can be classified as invasive or non‐invasive, or classified based on the degree of respiratory assistance the ventilator provides for the respiratory cycle, as such: fully controlled, partially assisted, unassisted.^2^ MV is used extensively in Intense Care Units (ICU's) and surgical settings. Indications for MV can be divided into three broad categories: (a) Reduced level of consciousness (eg, head trauma, drug overdose, anesthesia), (b) Respiratory failure (eg, ARDS, COVID‐19, COPD) and (c) Circulatory failure.^2^

In low‐income countries (LMIC's), the four most common indications requiring MV in ICUs are post‐surgical care, sepsis, trauma, and maternal peripartum or neonatal complications.^3^ A significant majority of these cases can be successfully managed with intervention involving a short course of MV. Widespread access to MV in a timely fashion can thus effectively be used to help patients in these settings and significantly improve outcomes.^3^

## 2 GLOBAL CHALLENGES

The Lancet Commission on Global Health in 2013^4^ highlighted the real possibility of reducing mortality rates of LMICs: to levels at which a “grand convergence” in health can be reached by 2035, to the level of current well‐performing Middle‐Income Countries. This in turn is predicted to yield proportionally substantial benefits to the economies of LMICs, with 11% of recent economic growth accounted by a reduction in mortality. The relative economic benefits exceed the cost of the initial financial outlay by a factor of 9 to 20 over the 2015 to 2035 period.^4^ The value of ventilators for both short and longer‐term use in low middle‐income countries (LMICs) is clear. However, the availability of ventilators is woefully short of demand. Data on ICUs, a proxy measure for hospital ventilator capacity in LMICs, suggest that capacity is extremely limited where it exists at all. More than 50% of LMICs do not have any data on ventilator/ICU capacity.^5^ This highlights the fragmentation and limited infrastructure present in these countries. It is also important to note that the burden of cardiorespiratory failure in LMICs may be under represented due to patients in whom intensive care was never initiated in the first place due to lack of availability of treatment.^6^ This urgent need is further highlighted by the globally unprecedented and escalating SARS‐CoV‐2 pandemic,^7^ for which MV has an essential role in treatment.^8^ As the pandemic progresses, ventilatory capacity will be completely overwhelmed in LMICs.^9^ Even high‐income countries such as the US, have struggled with access to ventilators as the prevalence of SARS‐CoV‐2 reaches overwhelmingly high rates.^10^ In fact, there is growing evidence to suggest that LMICs with a high SARS‐CoV‐2 prevalence have already been suffering such shortages, such as in India,^9^, ^11^, ^12^ which has recorded over 10.5 million cases and 150, 000 deaths, as of January 2021.^13^

Another vital factor to consider is the significant relative shortage of trained medical staff in LMICs. Estimates by the WHO highlight a global shortage of 4.3 million medical staff, including doctors, midwives, nurses, and other healthcare workers.^14^ Seventy‐five countries had fewer than 2.5 health workers per 1000,^14^ known as the minimum ratio required for basic health services. Most of these shortages are known to be in LMICs and this imbalance between the supply and demand of staff is further illustrated in the case of Africa; by 2009 estimates, 1.3% of the world's medical staff has been providing care for 25% of the global disease burden.^15^ Multiple reasons for such shortages exist, such as insufficient infrastructure for clinical education and weak regulation of education standards.^16^ Furthermore, “brain drain,”^14^, ^17^: emigration of skilled medical staff from LMICs to HICs which are perceived to have superior economies, working conditions, political stability, and more opportunities for career progression,^16^ alongside an aging workforce,^17^ are seen to have drastically exacerbated this issue. Shocking, life‐endangering situations in the African continent have been reported where health services have vanished overnight due to the sudden emigration of skilled workers.^14^ It is also thought that this phenomenon not only results in direct loss of labor supply in the health sector but also results in the consequential loss of future investments into training and education.^18^

This situation is further complicated due to healthcare imbalances present between urban and rural regions in LMICs. In many LMICs, much of the population inhabit rural areas. However, most large hospitals are located in cities, thereby reducing the population's accessibility to advanced healthcare.^19^ As a result, critically ill patients in rural and remote settings are treated in basic ward environments of regional medical facilities.^3^, ^5^ Even in those few rural regional hospitals which have ICUs, the number of beds available is often lower compared to urban ICUs.^6^ Furthermore, there is a significant shortage of skilled healthcare professionals capable of providing advanced levels of care beyond urban concentrations of provision.^20^ These factors have all contributed to rural and remote regions in LMICs having significantly poorer health outcomes, especially in the critically unwell, and a lower life expectancy compared to both urban regions in LMICs as well as higher‐income countries (HICs).^21^ The etiology of critical illness is also different between rural and urban settings. For instance, a common cause of severe illness in rural LMIC settings is acute neuromuscular paralysis induced by snakebite venom.^22^ Annually, it is estimated that 4.5 to 5.4 million people get bitten by snakes, of which 81 000 to 138 000 die from complications.^23^ Both mortality and morbidity are greatest in rural communities of LMICs, due to the factors described above.^24^ MV is a critical component of the care necessary for envenomation and failure to access such care results in significant mortality.^22^, ^24^

The literature clearly suggests that there is a real and urgent need for a simple, easily maintained, and operated mechanical ventilator or a respiratory support device to significantly reduce mortality in a wide range of conditions.

## 3 INFRASTRUCTURE FOR HEALTH SERVICES

Due to the limited healthcare infrastructure present in LMIC's, a significant amount of medical treatment is likely to occur in low resource settings. These settings are likely to have limited access to electricity and compressed oxygen and are likely to be in semi‐permanent structures. In these scenarios, portable respiratory support devices would be the most effective tool to provide ventilation to those who need it. The majority of current portable ventilators are complex and fragile devices that consist of sophisticated hardware and software. They require trained staff to calibrate, repair, and use the device. The cost of these devices range from 3300 to 13 500 USD and are therefore beyond the reach of many healthcare systems even if they could be powered and maintained reliably.^25^

### 3.1 Economic impact related to the age of the patient

Interestingly, in LMIC populations, younger patients are often disproportionately affected by critical illness compared to the developed world.^26^ Each death of a young patient, which otherwise could have been prevented through timely MV, would translate to the loss of many years of potential contribution to the LMIC's economy and productivity. Younger patients have reduced short‐term and long‐term mortality and morbidity than older patients after critical illness,^27^ with a greater likelihood for recovery and productivity. Thus, increased availability of MVs in LMICs, through the development of an economical and robust device, will not only benefit the health economy in these countries but will improve the overall prosperity by saving lives.^28^

### 3.2 Maternal mortality

A woman's lifetime risk of maternal mortality is 1 in 38 in sub‐Saharan Africa, compared to 1 in 5100 in Europe.^29^ This is despite the UN Millennium Development Goal 5, an effort by the World Health Organization (WHO), which aimed to reduce global maternal mortality by 75% and focused on regions with higher maternal mortality. Even though the global maternal mortality rate was nearly halved in these efforts, more than half of all maternal deaths worldwide still occur in Sub‐Saharan Africa,^30^ most commonly due to hemorrhage, pre‐eclampsia/eclampsia, or sepsis.^31^ According a recently published report, maternal and neonatal disorders is one of the top five conditions that has the potential for reduction in the disease burden. This reduction, in this group, can be related to 120 disability‐adjusted life years.^28^ A key factor that contributes to such devastating mortality rates is the severe shortage and accessibility of medical and surgical equipment^32^ and ventilators,^33^ further reinforcing the urgent need for accessible and low‐cost MVs.

### 3.3 Other critical scenarios

Simple mechanical ventilation would also provide a huge benefit in the management of road traffic collisions. LMICs are disproportionately affected by road traffic accidents, with 90% of global road traffic deaths occurring in these countries.^34^ Literature suggests that 68% of trauma admissions in the low‐income setting require intensive care support including ventilation.^35^ As previously established, there is a distinct lack of ventilator capacity in these settings, so increased availability of these devices would significantly improve mortality.

## 4 THE WAY FORWARD

This all highlights the need to provide a MV that is portable, robust, and financially viable in LMICs. The WHO specification for a simple portable ventilator is:

- Air compression
- Oxygen mixing capacity to an accuracy of 4%
- Can generate a FiO_2_ from 21% to 100%
- Can generate a tidal volume of 20 to 1000 mL (Medicines and Healthcare products Regulatory Agency, UK regulation is 400 +/− 10%)
- Can generate an I:E ratio of 1:2
- Can generate a respiratory rate of 10 to 60 bpm
- A test function to assess for air leaks
- A display of these parameters
- An alarm function for parameters that fall outside the normal range
- Easily cleaned.

These simple criteria differ from the more complex criteria for hospital ventilators and can be met by analog devices using basic technology such as a modified bag valve mask (BVM) or a high‐pressure blower.^36^

Modern portable ventilators, though effective, are expensive and therefore inaccessible in many parts of the world. The WHO criteria above needs to be fulfilled by a simple device which can be marketed for a significantly lower cost than existing devices. In addition, one needs to note that the range provided for tidal volumes and the respiratory rates in the WHO specification could be altered depending on where and how these devices are used. For example, a tidal volume range of 250 to 550 should be sufficient for adult use in certain emergency scenarios. In terms of respiratory rates, with an I:E of 1:1, a device which can go up to max 25 bpm could be sufficient in majority of adult cases.

Ideally, such a device would be locally manufactured and locally maintained to both mitigate against cost and logistical barriers as well as augment the local health economy. The use of such a device will need to be largely intuitive or at least require only fundamental training such that its use by a variety of healthcare providers is facilitated.

In conclusion, there is a clear and urgent need for simple, low‐cost, portable ventilators or respiratory support devices in LMICs, the availability of which would have a profound effect on healthcare and the health economy.

## CONFLICT OF INTEREST

All authors declare that they have no support or financial relationships from any organization for the submitted work.

## AUTHOR CONTRIBUTIONS

Conceptualization: Nachiappan Chockalingam.

Investigation: Nitish Nachiappan, Jung Moses Koo.

Methodology: Nitish Nachiappan, Jung Moses Koo.

Supervision: Nachiappan Chockalingam, Timothy Scott.

Writing – Original Draft Preparation: Nitish Nachiappan, Jung Moses Koo.

Writing – Review & Editing: Nachiappan Chockalingam, Timothy Scott.

All authors have read and approved final version of the manuscript.

## TRANSPARENCY STATEMENT

Professor Nachiappan Chockalingam affirms that the manuscript is an honest, accurate, and transparent perspective of all authors.

## Data Availability

This is a perspective of the authors and no experimental data were collected. The authors are happy to share information on the resources used for this paper, on request.
